# Efficacy and side effects of doxycycline *versus* minocycline in the three-dose melarsomine canine adulticidal heartworm treatment protocol

**DOI:** 10.1186/s13071-018-3264-z

**Published:** 2018-12-27

**Authors:** Molly D. Savadelis, Katherine M. Day, Jenna L. Bradner, Adrian J. Wolstenholme, Michael T. Dzimianski, Andrew R. Moorhead

**Affiliations:** 1 College of Veterinary Medicine, University of Georgia, Athens, GA USA; 2 Center for Tropical and Emerging Global Disease, University of Georgia, Athens, GA USA

**Keywords:** Canine heartworm disease, *Dirofilaria immitis*, *Wolbachia*, Doxycycline, Minocycline, qPCR

## Abstract

**Background:**

The American Heartworm Society currently recommends the use of a monthly macrocyclic lactone, a 28-day course of 10 mg/kg doxycycline BID, and the 3-dose protocol of melarsomine dihydrochloride for the treatment of canine heartworm disease. Doxycycline is necessary for the reduction of the bacterium *Wolbachia*, found in all heartworm life-stages. Previous price increases and decreasing availability prompted us to evaluate alternative tetracycline antibiotics, i.e. minocycline, for the reduction of *Wolbachia* during canine heartworm treatment.

**Methods:**

Thirty-two heartworm-positive dogs were randomized to receive 10 mg/kg or 5 mg/kg of either doxycycline or minocycline for 28 days BID, for a total of 8 dogs per experimental group. All dogs received 6 months of Heartgard Plus® (ivermectin/pyrantel) and the 3-dose protocol of 2.5 mg/kg melarsomine dihydrochloride. Blood samples were collected prior to the initiation of treatment, every 7 days throughout tetracycline treatment, and then monthly thereafter until the dog tested negative for the presence of heartworm antigen. DNA was isolated from circulating microfilarial samples and qPCR was performed on each sample.

**Results:**

A greater number of dogs in the 10 mg/kg doxycycline and minocycline treated groups experienced gastrointestinal side effects as compared to the 5 mg/kg doxycycline and minocycline treated groups. All eight dogs in the 10 mg/kg doxycycline-treated group tested negative for the presence of *Wolbachia* DNA by 28 days post-tetracycline treatment. A total of two dogs in both the 5 mg/kg doxycycline- and 10 mg/kg minocycline-treated groups and three dogs in the 5 mg/kg minocycline-treated group remained positive for the presence of *Wolbachia* DNA by the end of tetracycline treatment.

**Conclusions:**

No lung pathology was assessed in this clinical trial, therefore the clinical effect of the remaining *Wolbachia* DNA in the 10 mg/kg minocycline-, 5 mg/kg doxycycline- and 5 mg/kg minocycline-treated groups cannot be determined. Owner compliance in the proper administration of these tetracyclines may be impacted by the increased severe gastrointestinal side effects reported for the 10 mg/kg doxycycline- and minocycline-treated groups. We recommend that veterinarians prescribe the recommended 10 mg/kg doxycycline for canine heartworm treatment and reduce the dosage to 5 mg/kg in cases of severe gastrointestinal side effects in order to improve owner compliance in administration of medications.

## Background

The American Heartworm Society currently recommends the use of a monthly macrocyclic lactone, 28 days of 10 mg/kg oral doxycycline twice daily, and a 3-dose protocol of 2.5 mg/kg intramuscular injections of melarsomine dihydrochloride for the adulticidal treatment of canine heartworm disease [1]. The tetracycline antibiotic, doxycycline, is utilized to remove the obligate endosymbiont *Wolbachia*, which are essential for the long-term viability of the adult parasite [2, 3]. With the death of adult heartworms, large quantities of *Wolbachia* are released into the host triggering an inflammatory immune response which can lead to host lung arterial and parenchymal lesions [4]. The addition of doxycycline during melarsomine treatment has been documented to reduce the overall arterial and parenchymal lung lesion scores as compared to melarsomine-only treated dogs, presumably due to this reduction of *Wolbachia* [5, 6].

While the use of doxycycline has been documented to be beneficial in canine adulticidal heartworm treatment, the dosage and duration currently recommended have been extrapolated from the treatment of other rickettsial infections, such as *Ehrlichia canis* [7]*.* A related tetracycline, minocycline, has recently been evaluated against *Wolbachia* in *Onchocerca gutterosa*, demonstrating *in vitro* efficacy against this endosymbiont [8]. Additionally, minocycline has less protein binding than doxycycline, allowing for increased tissue distribution [9]. Despite this, the use of minocycline in canine adulticidal heartworm treatment has not been evaluated.

The effects of antibiotics against other filarial worms including *Brugia* species, *Onchocerca* species, *Wuchereria bancrofti* and *Litomosoides sigmodontis* have been evaluated both *in vitro* and *in vivo* [2, 10–12]*.* In some studies, timing in the administration of tetracyclines appeared to cause different effects against the developmental stages of the worm. Tetracycline treatment of *Brugia pahangi* in the gerbil *Meriones unguiculatus* lead to sex-ratio changes when administered during the late female fourth larval stage molt and had a prophylactic effect when administered during fourth larval stage and the early adult stage [3, 13]. Additional studies support the blocking of embryogenesis of filarial parasites and the potential role for preventing transmission of these diseases for mass drug administration [11, 14]. These data indicates that tetracycline may be effective as a treatment for human and animal filarial worms that contain *Wolbachia*.

With the fluctuating price and availability of doxycycline, an alternative tetracycline for the reduction of *Wolbachia* in canine adulticidal heartworm treatment is necessary [15]. In this study, the overall clinical effects and efficacy in the reduction of *Wolbachia* in circulating microfilariae throughout canine heartworm treatment was evaluated using doxycycline and minocycline at various dosages.

## Methods

### Study animals

The study protocol, CR-450, was approved by the University of Georgia’s Clinical Research Committee, the Hospital Board and the University of Georgia Research Foundation prior to the start of the study. This clinical trial utilized a total of 32 dogs with 4 experimental groups, and 8 dogs per group. Heartworm-positive dogs with circulating microfilariae present with no previous tetracycline or heartworm adulticidal treatment were enrolled. At the time of enrollment, dogs were randomized to receive either 10 mg/kg doxycycline, 5 mg/kg doxycycline, 10 mg/kg minocycline, or 5 mg/kg minocycline, PO BID for 28 days, according to a randomized block design at the time of enrollment. All dogs received the correct dosage of monthly Heartgard® Plus (ivermectin/pyrantel, Merial Limited, Duluth, GA, USA) for a total of 6 months, 28 days of tetracycline treatment according to group designation, and the 3-dose protocol of 2.5 mg/kg melarsomine dihydrochloride for the treatment of heartworm disease. Dogs received either Immiticide® (Merial Limited, Duluth, GA, USA) or Diroban™ (Zoetis Inc., Kalamazoo, MI, USA) according to the availability of these products.

### Microfilarial concentration and heartworm antigen

Blood samples were obtained prior to the initiation of treatment, every 7 days throughout tetracycline treatment, and monthly thereafter until the dog tested negative for the presence of heartworm antigen. Microfilarial concentrations and the presence of heartworm antigen were assessed at each time-point, and purified microfilariae were obtained for DNA isolation. The presence of heartworm antigen was evaluated using the DiroCHEK® Heartworm Antigen Test Kit (Synbiotics Corporation, Zoetis Inc., Kalamazoo, MI, USA) using serum samples [16]. Thick smears and modified Knott samples were prepared from 20 μl of anticoagulated blood and 1 ml of anticoagulated blood in 10 ml 2% formalin, respectively. Circulating microfilariae were isolated from whole blood using syringe filtration for DNA isolation. Whole blood containing microfilariae was lysed using a 0.2% saponin 0.85% NaCl solution for 10 min in a 37 °C water bath and then filtered through a syringe filter containing a 5.0 μm mesh filter to capture the microfilariae. Isolated microfilariae were stored in phosphate buffered saline (Boston BioProducts, Ashland, MA, USA) at -20 °C.

### Quantitative PCR

Relative quantitative PCR was performed to analyze the reduction of *Wolbachia* DNA to the amount of *Dirofilaria immitis* DNA from circulating microfilariae collected during tetracycline treatment. PCR was performed on each dog sample at each time point in quadruplicate using a Corning™ Axygen™ 96-well PCR microplate (Fisher Scientific 14-222-322, Axygen, Tewksbury, MA, USA) and a Stratagene Mx3000P Real-Time PCR System (Agilent Genomics, Santa Clara, CA, USA). Primers were generated for *Wolbachia ftsZ* (GenBank: AJ495000) and *D. immitis 18S* (GenBank: AF036638) DNA regions and Taqman® probes containing HEX and FAM fluorescent probes were created, respectively. *Wolbachia* amplicon primers included forward (5'-GCT GGT GCC TTA CCT GAT ATT-3') and reverse (5'-CCA CCC ATT CCT GCT GTT AT-3') to amplify a 110-bp fragment. *Dirofilaria immitis* amplicon primers included forward (5'-TGA GAA ACG GCT ACC ACA TC-3') and reverse (5'-GAT AAC CGG CCT CAT AGA GAA C-3') to amplify a 112-bp fragment. Taqman® probes included 5'-HEX-TCG ATT CTT CTG CTG CAC CTT TAC C-BHQ_1-3' for *Wolbachia ftsZ* and 5' FAM-TTC TGA GAT GGG TAA TTT GCG CGC-BHQ_1-3' for *D. immitis 18S*. Heartworm-positive, non-treated, control dog DNA samples were obtained at the same time points to compare natural fluctuations of *Wolbachia* relative to *D. immitis*.

### Tetracycline treatment side effects

During the 28-day tetracycline treatment regimen, each dog was observed every 7 days to obtain blood samples and weight measurements. Throughout this time period, owners were asked to report any abnormal behaviors or side effects. Gastrointestinal side effects reported such as vomiting, diarrhea, inappetence and weight loss were documented at each visit with the owner detailing the date and number of occurrences of each symptom. To subjectively score the severity of gastrointestinal side effects for each dog throughout tetracycline treatment, a score of 1 was added for each symptom reported for a maximum severity score of 4 and a minimum of 0. Severity scores were not increased if a symptom was reported more than once in order to prevent bias in inaccurate owner reporting.

### Statistical analysis

All data were analyzed using GraphPad Prism 7.04 (La Jolla, San Diego, CA, USA). Data generated in this study were analyzed by analysis of variance with a Tukey’s multiple comparison test and Mantel-Cox test.

## Results

### Gastrointestinal side-effects

**Fig. 1:**
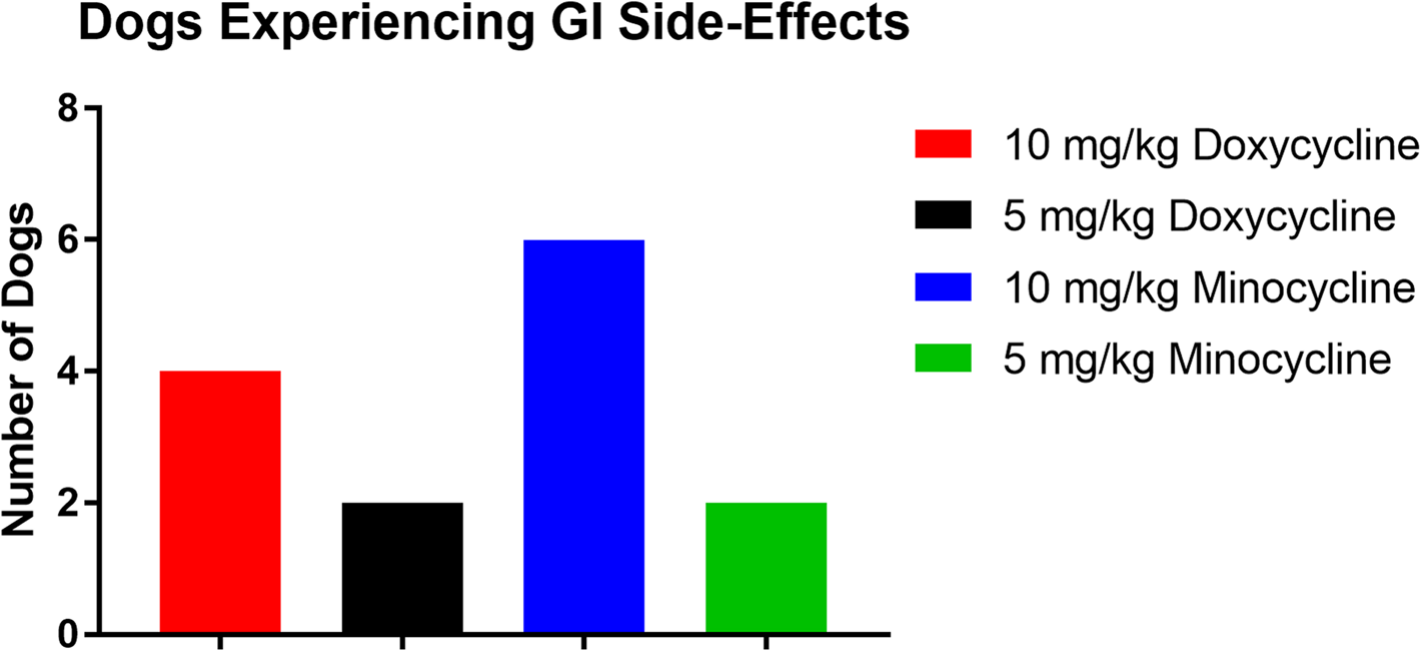
Number of dogs in each experimental tetracycline group in which the dog’s owners reported any gastrointestinal side-effects during antibiotic treatment. Side-effects included vomiting, diarrhea, inappetence and weight loss

**Fig. 2:**
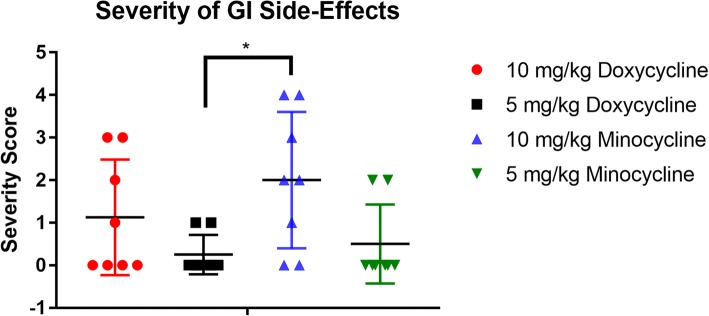
The relative severity of gastrointestinal side-effects reported by owners was generated by added a score of 1 for each side-effect reported such as vomiting, diarrhea, inappetence, or weight loss, with a maximum severity score of 4 if all side-effects were reported. If a side-effect was reported multiple times, this was only counted once. Severity scores are significantly higher in the 10 mg/kg minocycline group as compared to the 5 mg/kg doxycycline treated group (*F*_(3,28)_ = 3.549, *P* = 0.0278)

A greater number of dogs experienced gastrointestinal symptoms during tetracycline treatment in those dogs receiving 10 mg/kg doxycycline and minocycline as compared to 5 mg/kg doxycycline and minocycline (Fig. 1). This increase of reported gastrointestinal side effects was not statistically significant. Additionally, the gastrointestinal symptoms were more severe in the 10 mg/kg groups, with symptoms such as vomiting, inappetence, diarrhea and weight loss (Fig. 2). The severity score of dogs receiving 10 mg/kg minocycline was statistically significantly higher than the 5 mg/kg doxycycline group (*F*_(3,28)_ = 3.549, *P* = 0.0278). Three owners reported discontinuous administration of the antibiotics due to severe gastrointestinal symptoms, of which, one dog received 10 mg/kg doxycycline and two dogs received 10 mg/kg minocycline. These dogs experienced diarrhea, multiple reports of vomiting, inappetence and weight loss ranging from 0.9–4.5 kg within the 28-day treatment.

### Microfilarial concentrations

Prior to the initiation of treatment, all dogs had circulating microfilariae at concentrations ranging from 183 microfilariae/ml to 60,300 microfilariae/ml. The rate of microfilarial elimination observed was slower than FDA-labeled microfilaricidal products and was not significantly different between experimental groups. The monthly administration of ivermectin and pyrantel with either minocycline or doxycycline successfully eliminated the presence of circulating microfilariae in all dogs by study day 168, 3 months after the last melarsomine injection. A total of 93.7% of all study dogs were cleared of circulating microfilariae by study day 112, 1 month after the last melarsomine injection (Table 1). The concentration of circulating microfilariae prior to the initiation of treatment did not affect the length of time necessary for complete elimination.

**Table 1:** Percentage of dogs testing amicrofilaremic

Treatment group	Study day						
	0	28	56	84	112	140	168
Doxycycline (10 mg/kg)	0	25	50	75	87.5	87.5	100
Doxycycline (5 mg/kg)	0	12.5	25	75	87.5	100	100
Minocycline (10 mg/kg)	0	0	50	75	100	100	100
Minocycline (5 mg/kg)	0	12.5	37.5	87.5	100	100	100

*Notes*: Thick smears and modified Knott tests were performed prior to initiation of treatment, every 7 days throughout tetracycline treatment, and monthly thereafter until the dog tested negative for the presence of heartworm antigen. All study dogs tested negative for the presence of circulating microfilariae by study day 168, 3 months after the last melarsomine injection. All dogs received six months of oral Heartgard® Plus (ivermectin/pyrantel)

### Heartworm antigen

**Fig. 3:**
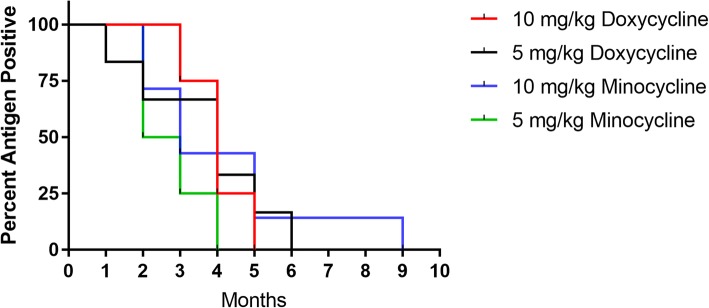
Study dogs were tested monthly for the presence of heartworm antigen using the DiroCHEK Heartworm Antigen Test Kit. In the 10 mg/kg minocycline treated group. One dog was tested at 6 months post-melarsomine but not at 7 and 8 months post-melarsomine due to scheduling conflicts. Therefore, this may have skewed this treatment group’s data

On average, study dogs tested negative for the presence of heartworm antigen by 4 months after the last melarsomine injection. All dogs tested negative for the presence of heartworm antigen by 5 months in the group receiving 10 mg/kg doxycycline, 6 months in the group receiving 5 mg/kg doxycycline, 9 months in the group receiving 10 mg/kg minocycline, and 4 months in the group receiving 5 mg/kg minocycline (Fig. 3). No statistically significant difference was observed in the length of time for testing heartworm antigen-negative between experimental tetracycline groups. Side effects reported post-melarsomine treatment included injection site swelling and pain, depressed mood and decreased appetite. One dog was diagnosed with severe pneumonia post-melarsomine treatment, which was successfully treated using BID Clavamox® (amoxicillin trihydrate/clavulanate potassium) for 7 days.

### Quantitative PCR

**Fig. 4:**
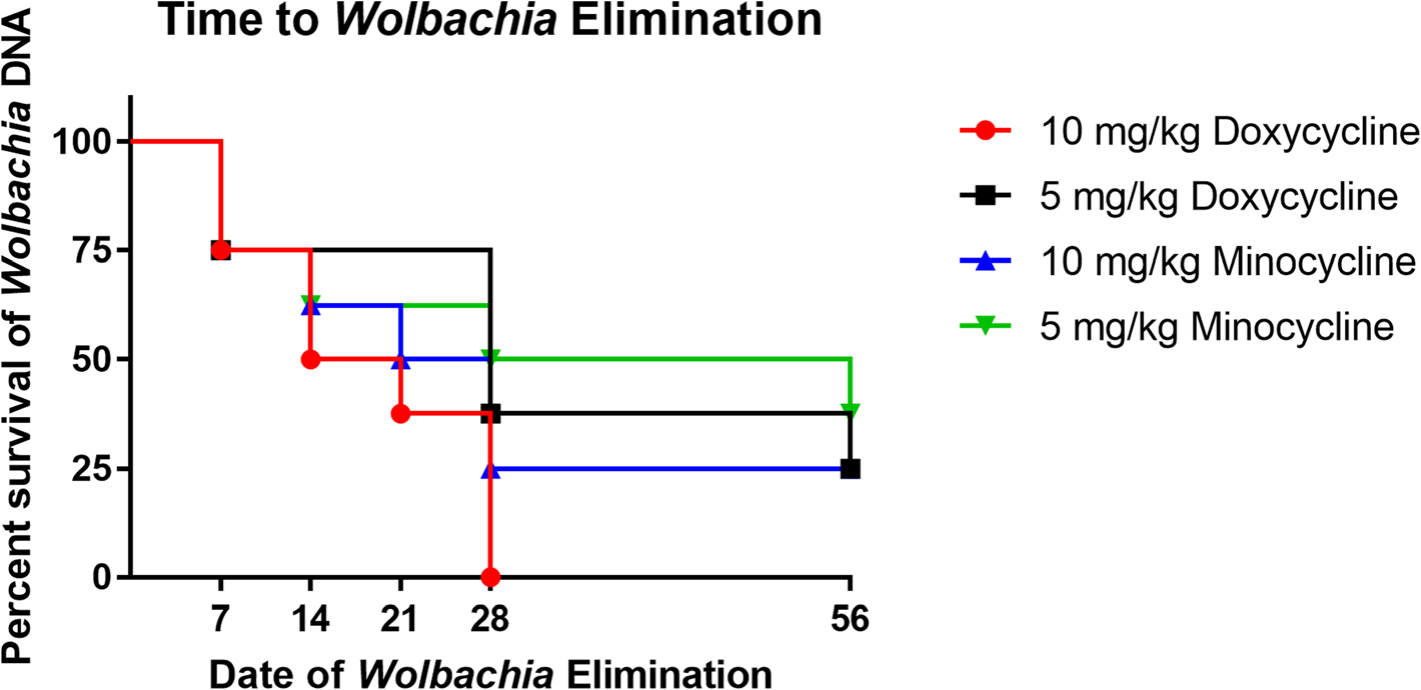
Quantitative PCR was performed on microfilariae isolated throughout tetracycline treatment for each study dog. Taqman® probes were generated for *D. immitis 18S* and *Wolbachia ftsZ* genes using FAM and HEX fluorescent probes, respectively. Samples were categorized as negative for the presence of *Wolbachia* DNA if at least three or more Ct values were ≥ 38 or no DNA was amplified

The non-treated control dog microfilarial samples collected had consistent quantities of *Wolbachia* DNA over a 28-day period by relative qPCR. Each microfilarial sample was tested for the presence of *Wolbachia ftsZ* DNA. Samples were designated negative for the presence of *Wolbachia* DNA when Ct values were ≥ 38 in 3 or more sample replicates or when no DNA was amplified. All eight dogs in the 10 mg/kg doxycycline treated group tested negative for the presence of *Wolbachia* DNA by 28 days post-tetracycline treatment. No other experimental groups had 100% of dogs testing negative for the presence of *Wolbachia* DNA. A total of two dogs in both the 5 mg/kg doxycycline and 10 mg/kg minocycline treated groups were still positive for the presence of *Wolbachia* DNA by the end of tetracycline treatment. Three dogs were still positive for the presence of *Wolbachia* DNA by the end of tetracycline treatment in the 5 mg/kg minocycline treated group (Fig. 4). No statistically significant difference was detected between groups in the number of dogs with *Wolbachia* DNA presence post-tetracycline treatment.

## Discussion

The American Heartworm Society currently recommends the administration of a monthly macrocyclic lactone, 28 days of 10 mg/kg doxycycline BID, and the 3-dose protocol of 2.5 mg/kg melarsomine dihydrochloride for the treatment of canine heartworm disease [1]. While no significant differences were found, the data suggests that the administration of 10 mg/kg doxycycline PO BID for 28 days eliminates the presence of *Wolbachia* DNA more efficiently than the 10 mg/kg minocycline and 5 mg/kg doxycycline and minocycline treated groups, supporting the use of 10 mg/kg doxycycline in canine heartworm treatment. The presence of circulating microfilariae without the amplification of *Wolbachia* DNA may indicate the successful elimination of live *Wolbachia* in microfilariae post-tetracycline treatment. While only two dogs in the 10 mg/kg minocycline-treated group remained positive for the presence of *Wolbachia* DNA by the end of tetracycline treatment, the use of minocycline during doxycycline shortages may be adequate for the reduction of *Wolbachia* during canine heartworm treatment.

Throughout this clinical trial, scheduling conflicts occasionally occurred in which owners were not able to bring study dogs in for blood sample collection for periods of time. Usually this only delayed blood sample collections for monthly testing, but in some instances monthly testing was entirely missed, such as in the instance of one study dog in the 10 mg/kg minocycline treated group. Blood samples were obtained at six months post-melarsomine for the presence of heartworm antigen and nine months post-melarsomine, but not at the seven and eight months post-melarsomine time points due to scheduling conflicts. This study animal was positive for the presence of heartworm antigen at the six months post-melarsomine time point and negative at nine months post-melarsomine. While the color intensity of the DiroCHEK® performed at six months was positive, this dog may have tested negative for the presence of heartworm antigen prior to nine months post-melarsomine, therefore biasing the antigen results for the 10 mg/kg minocycline-treated group.

Veterinarians should be aware of the severe gastrointestinal side effects reported for minocycline with six out of eight dogs reporting side effects, and four of the six dogs losing weight during antibiotic treatment. All owners were advised to fast their dogs prior to the administration of each antibiotic since this has been reported to increase the absorption of these antibiotics [9]. If the owner reported gastrointestinal side effects during treatment, they were then advised to administer the antibiotics with food to reduce gastrointestinal irritation. No concomitant medications were administered for gastrointestinal side effects.

Quantitative PCR has been used to evaluate the efficacy of doxycycline in reducing *Wolbachia* in canine heartworm disease previously. In one study, a total of 20 study dogs were experimentally infected with seven male and nine female 8-month-old adult heartworms by surgical transplantation. Five dogs were randomized into four experimental groups receiving either weekly prophylactic dosages (6 μg/kg) of ivermectin for 34 weeks, 10 mg/kg SID doxycycline PO during weeks 0–6, 10–12, 16–18, 22–26 and 28–34, a combination of ivermectin and doxycycline as stated above, or non-treated controls [17]. Adult heartworms were recovered at necropsy and stored at -80 °C for further molecular analysis. Quantitative PCR for the *Wolbachia ftsZ* and *D. immitis 18S* rDNA genes was performed using SYBR green fluorescent green dye. The median *Wolbachia*/*D. immitis* gene ratio decreased from 3.3 × 10^-3^ in the control group to 4 × 10^-5^ in the doxycycline-treated group adult females and from 5.1 × 10^-2^ in the control group to 1.8 × 10^-4^ in the doxycycline-treated group adult males [17]. This study demonstrated the efficacy of doxycycline in reducing *Wolbachia* concentrations in adult heartworms as well as the utility of qPCR in evaluating the reduction of *Wolbachia* in *D. immitis* life-stages.

The results obtained in this study for the elimination of *Wolbachia* DNA is comparable to other filarial worm data. In one study, the effect of various antibiotics including doxycycline and minocycline against *O. gutturosa* adult male mean motility and survival utilizing the MTT assay. After 40 days *in vitro*, minocycline and doxycycline at 5 × 10^-5^ M both had a 100% reduction in motility and a 93.7% and 93.0% inhibition of biochemical worm viability according to MTT [8]. Additionally, the quantitative PCR cycle threshold values (Ct) were determined for *B. pahangi* adult male and female worms isolated from gerbils for the *Wolbachia ftsZ* gene [18]. Gerbils were allocated to receive either 1.3% tetracycline in drinking water from 26–54 days post-infection, or remain non-treated controls. Baseline Ct values for non-treated control adult females ranged from 22.24–22.83 and adult males from 25.97–34.33. Ct values for tetracycline-treated adult females was 41.84 and adult males ranged from 43.80 - negative for the amplification of *Wolbachia* DNA [18]. Quantitative PCR results for the *D. immitis* microfilariae in the present study resulted in 62.5% of dogs testing negative for the presence of *Wolbachia* DNA by 28 days of tetracycline treatment in the 5 mg/kg minocycline treated group, 75% of dogs testing negative in the 5 mg/kg doxycycline and 10 mg/kg minocycline treated groups, and 100% in the 10 mg/kg doxycycline treated group.

Despite only sampling circulating microfilarial samples from treated dogs in this study, this clinical trial data may be a representation of the reduction of *Wolbachia* concentrations in adult heartworm populations. While clinical trials may offer more cost-beneficial options for quality research studies as compared to experimentally infected research animals, these studies have some scientific limitations. This study relied on owners to correctly administer the tetracyclines analyzed and to honestly report missed dosages and side effects during treatment. Improper dosage and administration of these antibiotics may have biased the qPCR data collected for each experimental group in this study. While experimental infections and controlled antibiotic administration may control for potential biases due to owner compliance, clinical trials may more closely resemble field data variability and conditions. Additionally, the age of each heartworm infection was unknown, therefore, differences in age of infection and its potential effect on *Wolbachia* concentrations may have influenced the qPCR data for individual study animals. Experimental heartworm infections can eliminate any potential bias in age of infection as well as improper administration of antibiotics. Further experimental studies need to be performed to quantify the exact *Wolbachia* reduction in adult heartworms during tetracycline treatment, as well as the effect of respective *Wolbachia* concentration reductions on lung pathology during canine heartworm treatment.

## Conclusions

With the complete elimination of *Wolbachia* DNA in the 10 mg/kg doxycycline treated group, the authors recommend administering 10 mg/kg PO BID doxycycline for canine adulticidal heartworm treatment. If severe gastrointestinal side effects are reported, veterinarians can determine if reducing the dose to 5 mg/kg PO BID would increase owner compliance in the administration of this medication. This study cannot conclude on the possible pathological effects of the remaining *Wolbachia* DNA present in the 10 mg/kg minocycline group and 5 mg/kg doxycycline and minocycline groups.
